# Using Tectona Grandis Biomass to Produce Valuable Adsorbents for Pesticide Removal from Liquid Effluent

**DOI:** 10.3390/ma15175842

**Published:** 2022-08-24

**Authors:** Isabel Pestana da Paixão Cansado, Paulo Alexandre Mira Mourão, Cristóvão Ramiro Belo

**Affiliations:** 1 MED—Mediterranean Institute for Agriculture, Environment and Development & Change—Global Change and Sustainability Institute, Departamento de Química e Bioquímica, Escola de Ciências e Tecnologia, Universidade de Évora, Rua Romão Ramalho nº 59, 7000-671 Évora, Portugal; 2 LAQV-Requimte, Departamento de Química e Bioquímica, ECT, IIFA, Universidade de Évora, Rua Romão Ramalho nº 59, 7000-671 Évora, Portugal; 3 Departamento do Ensino de Química, Faculdade de Educação, Artes e Humanidades, Universidade Nacional Timor Lorosa’e, Rua Jacinto Cândido, Dili 314, Timor-Leste

**Keywords:** Tectona Grandis, East Timor, activated carbon, chemical activation, pesticide removal, circular economy

## Abstract

This work presents a first approach concerning the valorization of Tectona Grandis tree by-products, from East Timor through their transformation into high activated carbon (AC) by chemical activation with KOH and K_2_CO_3_. The better ACs, Teak-KOH-1-1-700 and Teak-K_2_CO_3_-1-2-700, presented a high A_BET_ (995 and 1132 m^2^·g^−1^) and micropore volume (0.43 and 0.5 cm^3^·g^−1^), respectively. Both ACs were tested on the removal of four pesticides, from the liquid phase. Both ACs performed better than existing commercial types, presenting a maximum adsorption capacity of 1.88, 1.67, 1.10 and 0.89 mmol·g^−1^, for 4-chloro-2-methylphenoxyacetic acid, 2,4-dichlorophenoxyacetic acid, diuron and atrazine, respectively. Pesticide adsorption from diluted and concentrated solutions confirms that diffusion is the limiting factor. The possibility of implementing a production unit for ACs in East Timor is very promising for that country. It presents an opportunity for job creation, biomass waste reduction and a contribution to environmental sustainability, thereby following the principles of a circular economy.

## 1. Introduction

Developing countries, such as East Timor, cannot afford to not value logging by-products by transforming them into valuable materials to increase their incomes. The disposal of agricultural by-products, frequently classified as waste, often involves burning, through an energy conversion. The valorization of biomass subproducts through its transformation into activated carbons (ACs), which can be used, regenerated and reused for the removal of pesticides from liquid effluents, can contribute to waste reduction and local economic development [1,2].

Globally, there is a growing demand for activated carbon, for a variety of uses [3] but their application in water treatment and purification comes at the top of the list [4]. ACs were successfully tested for the removal of natural organic matter, pharmaceuticals, phenolic compounds, pesticides and heavy metals, among others [5,6,7,8]. The most commercially available ACs are relatively expensive and are produced from non-renewable sources. Biomass subproducts are excellent precursors for AC production given their high availability and high carbon content [2,9,10].

Approximately 34% of East Timor is covered by Tectona Grandis (Teak) trees that grow freely throughout Southeast Asia [2,11,12]. Due to its excellent properties, this wood has several applications, which, consequently, generates a large number of subproducts, making the cost of ACs prepared from biomass materials very low compared with the cost of commercially produced activated carbon [13].

Mashkoor and his co-authors reported the successful use of Tectona Grandis sawdust as a very low-cost adsorbent for the removal of crystal violet from synthetically contaminated wastewater [14]. Previous work by the same group allowed the valorization of subproducts from Tectona Grandis through their conversion into AC, by physical activation with carbon dioxide [10]. The transformation of biomass subproducts into ACs results in a reduction in their price and converts unwanted biomass subproducts into valuable adsorbents [15,16], this being in line with circular economy principles [1,10].

The literature abounds with papers reporting the use of different synthetic and natural waste and includes a diversity of agricultural subproducts, as a precursor for AC production. Mohammed and Tanweer’s review, in 2018, concerning the utilization of wood biomass as a sustainable precursor for AC production, revealed that these precursors allow adsorbents with well-developed physical and textural characteristics to be obtained [17]. The use of Tectona Grandis subproducts for the production of ACs was only referenced by a few works [10,18,19,20,21]. However, the present study is the first time that Tectona Grandis waste was used to obtain ACs by chemical activation with KOH and K_2_CO_3_.

Generally, experimental conditions, such as activation time, activating agent and activation temperature, play an important role in the development of the porous structure. It is agreed that ACs obtained after a chemical process, exhibit a high yield, good thermal stability, a large surface area and a well-developed porous structure, with a wide mean pore size and different surface functional groups [22]. It is still necessary to add the possibility of regeneration and successive reuse to their high performance in removing pollutants from the gaseous or liquid phase [23].

From another perspective, in developing countries, such as East Timor, the increasing need for food requires frequent use of pesticides in agricultural plantations, which in turn causes the contamination of soils and watercourses. The strategic plans of the Government of East Timor, for a period between 2014 and 2020, included the provision and incentive for the use of pesticides and fertilizers as a means to increasing agriculture production [24].

Recent work, however, reported that, due to the presence of pesticides, pollution exists worldwide. Moreover, an area of 2.5 billion hectares of agricultural land was already identified as being at risk of pollution by more than one active ingredient present in pesticides [25]. Many pesticides are recalcitrant [26] so, to ensure soil sustainable utilization, liquid streams must be treated, thereby allowing the removal of pesticides, some of which are classified as emerging pollutants.

Among the various techniques used for pollutant removal from liquid effluent, adsorption by different adsorbent materials deserves merit for its performance and ease of implementation [27,28], with emphasis on the use of AC.

The main objective of this work was the production of high porous ACs from Tectona Grandis sawdust by chemical activation using KOH and K_2_CO_3_ at different temperatures, with high potential applications for pesticide removal from liquid effluent. In this work, emphasis was given to the removal of 4-chloro-2-methylphenoxyacetic acid, 2,4-dichlorophenoxyaceticacid, atrazine and diuron, from liquid effluent; this is because these pollutants are commonly found in soil and watercourses due to their frequent use as post-emergent herbicides. The pesticides studied are included on the list of pesticides whose presence must be checked on a drinking water, in Portugal [29]. The removal of these pesticides from liquid streams is imperative since they have a half-life in soil ranging from 15 to 50 days, depending on the environmental conditions [28,30].

## 2. Materials and Methods

### 2.1. Preparation of Activated Carbon from Tectona Grandis

Activated carbons with a well-developed micropore volume were produced from Tectona Grandis sawdust (Teak), by chemical activation with KOH and K_2_CO_3_, at different temperatures. Several pieces of wood from the Tectona Grandis tree were transported directly from East Timor. The wood pieces were crushed, using a blender, into smaller particles (0.2–0.3 mm diameter) in our laboratory and used without any treatment.

Two amounts of 4 g of Teak sawdust were placed in two separate containers and dry-impregnated with either potassium hydroxide (KOH) or potassium carbonate (K_2_CO_3_). Based on previous work and the data presented in a review by Heidarinejad and co-workers, physical mixing of the precursor with alkali salts was performed. This produces ACs with a higher porosity than that of ACs produced by the aqueous impregnation method [3]. The mixtures were placed in the furnace and submitted to a heating rate of 10 K min^−1^ until a final temperature between 723 and 973 K was achieved. Chemical activation was performed under a nitrogen flow of 70 mL min^−1^. The previously defined heating conditions were used for comparative purposes with ACs prepared earlier by physical activation with CO_2_, using the same precursor [10]. Some ACs presented in this paper were produced in a small horizontal furnace, from Thermolab, and others were prepared in a semi-industrial rotating horizontal tubular furnace, High Temp Technology, TR–334/2018, from Thermolab, constructed for this purpose. The use of a semi-industrial furnace allows AC production to be scaled up to a semi-industrial level. The semi-industrial furnace enables the production of 100 times more AC, at a time, than that obtained using the laboratory furnace. The experimental conditions used in both furnaces (activation temperature, heating rate, nitrogen flux) were similar. The only difference was to ensure that the inside of the semi-industrial rotating horizontal tubular furnace was in an inert environment before starting the chemical activation process. The ACs were cooled to room temperature and removed from the furnace under a constant nitrogen atmosphere. The ACs were then washed repeatedly with distilled water, until the washing water had a similar pH to the distilled water. After the optimized experimental conditions, the yield and textural characteristics of the ACs produced in both furnaces were comparable, so their subsequent use was undifferentiated. The following designation was assigned to the produced ACs: Teak-KOH-1-1-700 was the abbreviation for the precursor: 1-1 referred to the ratio of precursor/activating agents used on the mixture, KOH referred to the activating agent used and 700 referred to the chemical activation temperature, in Celsius.

### 2.2. Structural and Chemical Characterization

The ACs produced were fully characterized using different methods commonly used for this purpose, such as scan electronic microscopy (SEM), nitrogen adsorption at 77 K, Fourier transformation infrared spectroscopy (FTIR), elemental analysis (CHNS and O_2_) (EA) and determination of the pH at point of zero charge (pH_pzc_).

#### 2.2.1. Textural Characterization

N_2_ adsorption isotherms were obtained on all ACs at 77 K, on Quadrasorb gas adsorption manometric equipment (Quantachrome Instruments), to evaluate their surface area, pore volume and mean pore size. The conditions used can be found in previous work [29]. The acquired data were analyzed using the Dubinin–Radushkevich (DR) equation and the Brunauer–Emmett–Teller (BET) and alfa-s (α_s_) methods. The surface of the ACs was analyzed by SEM–EDX, using a Quanta 3D FEG-FEI, model HITACHI S-4800, with a Bruker Quantax EDS System.

#### 2.2.2. Chemical Characterization

To identify the predominant functional groups present in the external surface area of the ACs, FTIR spectroscopy was used. The spectra were recorded using a Perkin Elmer Spectrum Two FTIR Spectrophotometer, by the KBr disc method. The relationship between the ACs and dispersing agent (KBr) was 1 to 500. The spectra were obtained between 4000 and 450 cm^−1^ by overlapping 20 scans, with a resolution of 4 cm^−1^.

The acidic/basic character of the ACs was evaluated based on the determination of the pH at the point of zero charge (pH_PZC_). Suspensions containing a ratio of AC mass to volume of sodium nitrate solution (0.1 mol L^−1^ NaNO_3_) higher than 7% (m/v) were carefully prepared. Suspensions were continuously stirred for 48 h in an automatic bath, at a temperature of 298 K; the pH of the filtrates was then measured. The pH value was identified as pH_pzc_.

The CHNS composition was achieved using a CNSH-O Elemental Analyzer (Eurovector, series EuroEA 3000) under AC combustion in a pure O_2_ atmosphere. The gases resulting from the combustion of the ACs were measured and compared to the values obtained with reference standards (sulfanilamide), allowing the determination of the elemental composition of S, C, H and N. The O_2_ was quantified separately. The reported values are the mean values of a set of analyses made in triplicate.

### 2.3. Pesticide Removal from the Liquid Phase

The pesticides selected for the studies were 4-chloro-2-methylphenoxyacetic acid (MCPA), 2,4-dichlorophenoxyaceticacid (2,4-D), chloro-3-ethylamine-5-isopropylamino-2,4,6-triazine (atrazine—At) and (3-(3,4-dichlorophenyl)-1,1-dimethylurea) (diuron—D), all purchased from Sigma–Aldrich, Lisboa, Portugal. The pesticide adsorption isotherms were obtained from a single component system, using a static method. In the present work, 10.0 mg of an AC was added to 25.0 mL of the aqueous solutions with known concentrations of pesticide. The adsorption was completed after an equilibrium time of 24 h. The residual pesticide concentration was assessed by UV/visible spectrophotometry using a Nicolet Evolution 300 UV-Vis Spectrophotometer. The quantification was performed at wavelengths of 228 and 280 nm for MCPA; 230 and 285 nm for 2,4–D; 264 nm for atrazine; and 254 nm for diuron. The four pesticides were quantified by employing an external pattern, obtained at an appropriate wavelength. The pesticide amount adsorbed (Q_ads/_mmol·g^−1^) by each AC was assessed using the difference between the initial concentration (C_0_) and the remaining concentration in solution (C_eq_), divided by the mass of AC used and considering the volume of solution used. The effect of the solution pH, temperature and contact time on the pesticide removal was also evaluated. In equilibrium adsorption experiments, higher concentrations than typically used for environmental applications are used; this is because these ACs are mostly used in emergencies, where high amounts of pesticides are spilt.

The data were analyzed using the Langmuir and Freundlich equations, allowing the monolayer capacity (n_mL_), Langmuir constant (K_L_) and Freundlich constants (K_F_ and n_F_) to be obtained.

## 3. Results and Discussion

### 3.1. Activated Carbon Production by Chemical Activation

A series of ACs was produced from Tectona Grandis (Teak) sawdust by chemical activation with KOH and K_2_CO_3_, at temperatures varying between 723 and 973 K. Some ACs presented in this paper were produced in a small furnace, while others were prepared in a semi-pilot furnace. As the textural characteristics of the ACs produced under optimized experimental conditions were comparable, their use was undifferentiated. The use of a semi-pilot furnace enables the production of a large amount of ACs in each batch to be used on large scale. The data presented in Table 1 and Table 2 demonstrate that the yield of the ACs produced at 973 K was slightly higher when K_2_CO_3_ was used, compared with the use of KOH. Nevertheless, with both activation agents, the yield decreases with increasing temperature.

A yield reduction was observed with a decrease in the ratio between the precursor/activating agent (Teak-KOH-2-1-700/Teak-KOH-1-2-700 and Teak-K_2_CO_3_-2-1-700/Teak-K_2_CO_3_-1-2-700), independently of the activating agent used. The use of excess activating agent promoted an excessive attack on the precursor, thus reducing the amount of ACs produced and, consequently, the yield of the process. The relationship between the precursor and the activating agents of 1:1 was considered the optimal value, so the results will be discussed simultaneously whenever possible.

### 3.2. Activated Carbon Characterization

The activated carbons were characterized based on nitrogen adsorption, held at 77 K. Table 1 and Table 2 display the textural characteristics of ACs produced by chemical activation with KOH and K_2_CO_3_, respectively. The data were obtained from analysis of the isotherms (Figures S1 and S2, Supplementary Materials) based on the BET, DR and α_s_ methods.

The apparent surface area and total pore volume increased with an increase in the activation temperature, from 723 to 973 K. Chemical activation is described as requiring a lower temperature than that used in physical activation; however, the ACs produced at 723 K with both activating agents presented a very low A_BET_ (Teak-KOH-1-1-450, 170 m^2^·g^−1^; Teak-K_2_CO_3_-1-1-450, 203 m^2^·g^−1^, respectively) when compared with similar ACs prepared at 973 K (Teak-KOH-1-1-700, 995 m^2^·g^−1^; Teak-K_2_CO_3_-1-1-700, 962 m^2^·g^−1^, respectively). On the N_2_ adsorption isotherm obtained from Teak-KOH-1-1-450 and Teak-K_2_CO_3_-1-1-450, the desorption branches for low pressures were not coincident with the adsorption branches. This phenomenon is characteristic of the presence of narrow micropores, in which, at high pressure, nitrogen molecules can enter but have difficulty leaving during the desorption step.

In the literature, it is common to find ACs prepared by chemical activation at a temperature lower than 873 K [15,33]. However, a compromise between the activation temperature and the desired ACs textural characteristics is essential. Previous results demonstrate that a temperature of 973 K is crucial to obtain ACs with a well-developed porous structure, this being a necessary condition for their application in pollutant removal from the liquid phase. Nevertheless, the use of higher temperatures was reported in a review paper concerning rice husk chemically activated with NaOH, coconut shell and bamboo chemically activated with KOH at 1123 K and rice-husk ash chemically activated with K_2_CO_3_ at 1273 K [3].

The influence of the ratio between precursor and activating agent on the ACs produced at 973 K presented a similar behavior for both KOH and K_2_CO_3_. The ACs produced with a precursor/activating agent ratio of 1:1 were those that presented the most developed textural characteristics. Using a ratio between precursor/activating agent of 2:1, the amount of activating agent was insufficient for complete activation of the precursor. In this situation, part of the precursor underwent a process similar to the carbonization process, reflected by a higher yield from the process (24.1% for Teak-KOH-2-1-700, 29.9% for Teak-K_2_CO_3_-2-1-700, 16.7% for Teak-KOH-1-1-700 and 24.0% for Teak-K_2_CO_3_-1-1-700). This led to ACs with an A_BET_ and porous volume significantly lower than those mentioned above with a ratio of precursor/activation agent of 1:1. The narrow porosity of Teak-KOH-2-1-700 and Teak-K_2_CO_3_-2-1-700 was also identified by the presence of a hysteresis cycle on the nitrogen adsorption isotherm, similar to those present in the ACs prepared at lower temperatures (Teak-KOH-1-1-450 and Teak-K_2_CO_3_-1-1-450). However, with Teak-KOH-2-1-700, the DR graphical representation presented a negative deviation from linearity, which confirms that the value of L_0_ = 3.05 nm, seen in Table 1, is not realistic. On the other hand, with a ratio of 1:2, the amount of activating agent seems excessive and promotes a marked activation of the precursor. The excess activating agent leads to the dehydration and destruction of the pore structure on the AC surface and conversion into larger pores, which then leads to the reduction in AC yield (Teak-KOH-1-2-700, 17.5%; Teak-K_2_CO_3_-2-1-700, 20.2%). The A_BET_ and pore volume of Teak-K_2_CO_3_-1-2-700 (A_BET_ 1132 m^2^·g^−1^, V_s_ 0.50 cm^3^·g^−1^) were higher than those obtained for Teak-K_2_CO_3_-1-1-700 (A_BET_ 962 m^2^·g^−1^, V_s_ 0.39 cm^3^·g^−1^). K_2_CO_3_ is considered a non-hazardous chemical, is not deleterious and is frequently used as a food additive [30]. However, the ratio of precursor/activating agent of 1:1 was used for comparative purposes and as means of reducing the production cost.

Both activating agents act in very similar way, but a comparison between the A_BET_ and total volumes of the ACs, obtained under the same experimental conditions, demonstrates that K_2_CO_3_ is more effective in the activation of Tectona Grandis sawdust. The same conclusion was achieved by Heidarinejad and his co-workers, based on a review of different studies concerning the chemical activation of a multiplicity of precursors and activating agents [3].

**Figure 1:**
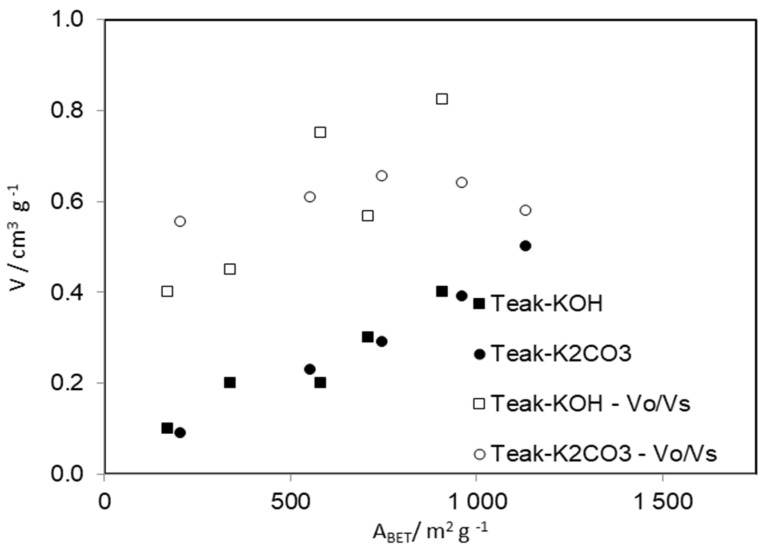
Total volume and reduced volume of the ACs prepared from Teak, by activation with KOH or K_2_CO_3_, at 973 K, as a function of the apparent surface area (A_BET_).

Figure 1 presents the total volume and reduced volume (volume of micropores (V_o_)/volume total (V_s_)) of the ACs prepared from Teak, by chemical activation with KOH and K_2_CO_3_, as a function of the apparent surface area (A_BET_). The total volume correlates very well with the apparent surface area and the reduced volume (V_o_/V_s_) correlates well with the A_BET_ in the ACs prepared with KOH, but the same tendencies were not observed in the ACs prepared with K_2_CO_3_.

In the ACs produced with K_2_CO_3_, there was an increase in the total porous volume, accompanied by the development of larger pores and also by the appearance of narrow pores. The ACs produced with K_2_CO_3_ presented slightly higher A_BET_ values when compared with the ACs prepared under the same experimental conditions with KOH, but presented a narrower average pore size. Some of the ACs produced in this work presented textural characteristics similar to those found in the literature. Castro prepared ACs from coffee grounds by chemical activation with K_2_CO_3_; the resulting ACs presented a surface area of 950 m^2^·g^−1^, a total volume of 0.45 cm^3^·g^−1^ and a micropore volume in the order of 0.38 cm^3^·g^−1^ [34]. Yuen and his co-workers produced ACs from *Enteromorpha proliferates* by chemical activation with K_2_CO_3_; these ACs presented a surface area of 2395 m^2^·g^−1^, a total volume of 1.80 cm^3^·g^−1^ and a mean pore size of 2.97 nm [35]. Based on their textural characteristics, Teak-K_2_CO_3_-1-1-700 (A_BET_ 962 m^2^·g^−1^, V_s_ 0.39 cm^3^·g^−1^) and Teak-KOH-1-1-700 (A_BET_ 996 m^2^·g^−1^, V_s_ 0.43 cm^3^·g^−1^) were chosen to undergo complete chemical characterization. Their excellent textural characteristics also led to their performance in the removal of pesticides from the aqueous phase.

The chemical characteristics of the ACs were evaluated by the determination of the pH at the point of zero charge (pHpzc), elemental analysis, SEM, EDX and FTIR. The ACs prepared from Tectona Grandis sawdust, at different temperatures, presented a moderate basic character, confirmed by an analogous pHpzc close to 8.5, as shown in the examples in Table 3. The ACs presented a carbon content superior to 80% and a small quantity of H and S; the nitrogen content was not quantifiable. The percentage of O_2_, included in Table 3, was obtained by EDX analysis, so the global percentage of the elements does not total 100%. From the EDX analysis (Figures S3 and S4, Supplementary Materials), the potassium was an external heteroatom present on the ACs, probably coming from the activating agent.

**Figure 2:**
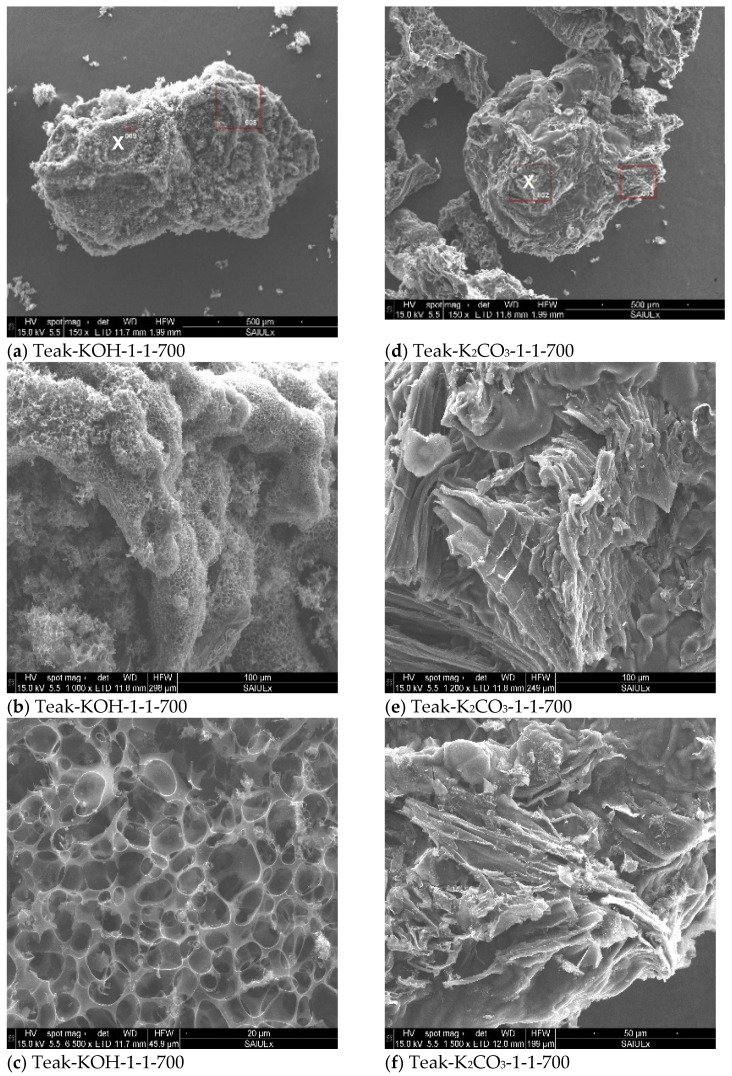
Representative SEM images obtained from Teak-KOH-1-1-700 and Teak-K_2_CO_3_-1-1-700. The images (**b**,**c**) are the amplifications of the points identified with an X, on image (**a**). On the images, each mark corresponds to 500 µm on the images (**d**), 100 µm on the images (**e**), 20 µm Teak-KOH-1-1-700 (**c**) and 50 µm Teak-K_2_CO_3_-1-1-700 (**f**).

The external area was analyzed by SEM and representative images are shown in Figure 2. It was noted that ACs produced by chemical activation with both activating agents at 973 K presented a well-developed porous structure. As reported earlier, the activation with K_2_CO_3_ was more effective, leading to the destruction of the Teak unit cells, illustrated for Teak-KOH-1-1-700 and Teak-K_2_CO_3_-1-1-700 in Figure 2c,f.

**Figure 3:**
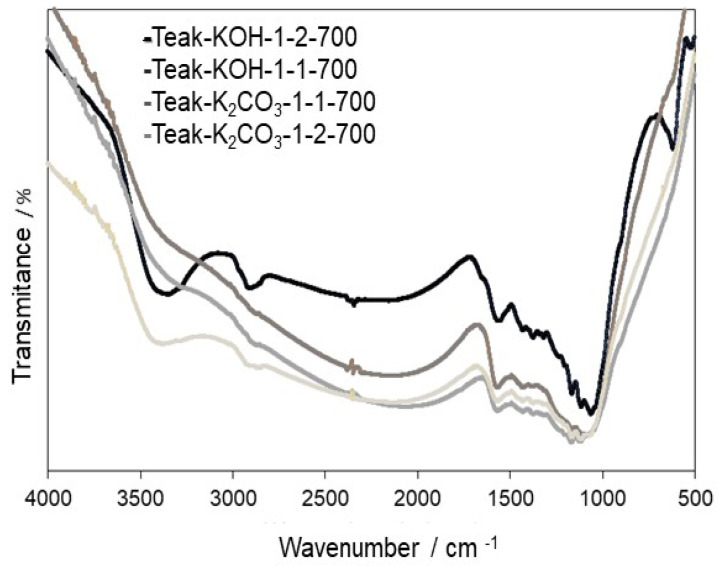
FTIR spectra of ACs prepared from Teak sawdust with KOH and K_2_CO_3_, at 973 K.

The FTIR spectra shown in Figure 3 show a set of characteristic bands present on the ACs prepared from lignocellulosic precursors, by chemical activation with KOH and K_2_CO_3_ [36]. The spectra of the four ACs seem similar, but on both ACs prepared with a ratio of 1:2 for the activating agent and precursor (Teak-KOH-1:2-700 and Teak-K_2_CO_3_-1:2-700), the presence of two bands around 3400 cm^−1^, and between 2800 and 3000 cm^−1^, stand out. These bands confirm the presence of an intense activation on the precursor, which leads to the emergence of new functional groups regardless of the activating agent used. The band at 3400 cm^−1^ can be attributed to the O–H group, in stretching mode, or the presence of NH groups; the contribution of these two functional groups may be overlapping.

Nitrogen, however, was not identified on these ACs, so these bands were assigned to the O–H group. The band around 2800 and 3000 cm^−1^ was assigned to the C–H connection on a CH_2_ or CH_3_ group, and the contribution of the OH, in a carboxylic group [28].

The band between 1510 and 1650 cm^−1^, common in all spectra, can be attributed to the presence of C=C or C=O groups in an aromatic ring. The bands of lower intensity identified between 1300 and 1560 cm^−1^ may be due to the contributions of vibration and flexion in the plane of the C–H bond. The band identified at 1400 cm^−1^ could be attributed to the S=O symmetric stretching frequency of organic sulphates. The presence of symmetrical deformations of C–O in a phenolic group could be identified by the band around 1150 cm^−1^, but this is not shown in Figure 3.

### 3.3. Pesticide Removal from the Aqueous Phase

In the aqueous phase, the adsorption capacity of the ACs for different pesticides with an aromatic ring in their structure, such as MCPA, 2,4-D and diuron, depends on numerous factors, and such complexity represents a challenge for researchers.

In this work, the pH of the solution containing the pesticide, where the AC was placed in contact with the pesticide, contact time, temperature and textural characteristics on pesticide removal from an aqueous media was evaluated.

Concerning the influence of the pH of the solution for MCPA and 2,4-D, higher adsorption capacities were achieved, with a pH of around 3, and agree with the results obtained by Spaltro et al., 2021 [37]. For atrazine and diuron, the best performances were achieved at a pH of around 7. In all four pesticides, the adsorption equilibrium of the removal process was achieved after a contact time of less than 24 h.

**Figure 4:**
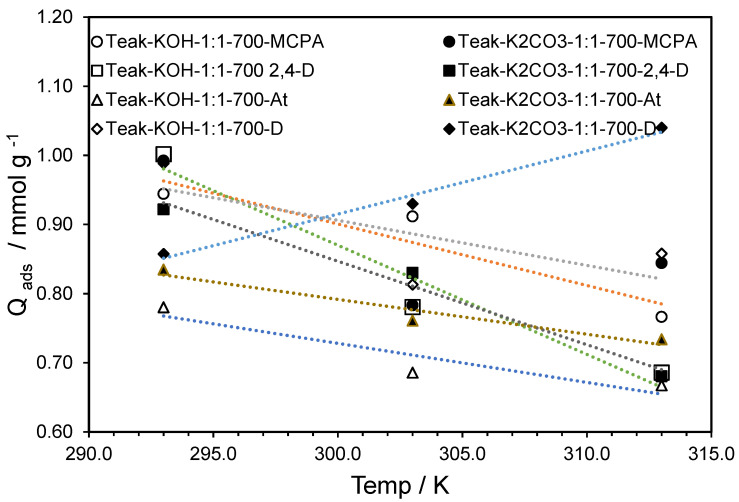
Temperature influence on the maximum adsorption capacity of Teak-KOH-1-1-700 and Teak-H_2_CO_3_-1-1-700, for the four pesticides.

Figure 4 shows that in the range of temperatures between 291 and 333 K, for both ACs, the adsorption of MCPA, 2,4-D and atrazine was an exothermic process. The influence of temperature on diuron adsorption was not conclusive.

The increase in temperature increases the MCPA and 2,4-D solubility in water, which may result in an adsorption decrease. Based on these results, adsorption of MCPA and 2,4-D is expected to be more effective in colder seasons. However, Derylo-Marczewska and co-workers found an increase in adsorption for the MCPA and 2,4-D adsorption on ACs Filtrasorb 300, with the temperature increase, which indicates an endothermic process. The trend was not very pronounced and, in some cases, may raise doubts; the authors explain this tendency by the enhanced mobility of 2,4-D and MCPA ions from the bulk solution near the adsorbent surface to an increase in the intraparticle diffusion and the creation of new active sites on the adsorbent surface [38].

**Figure 5:**
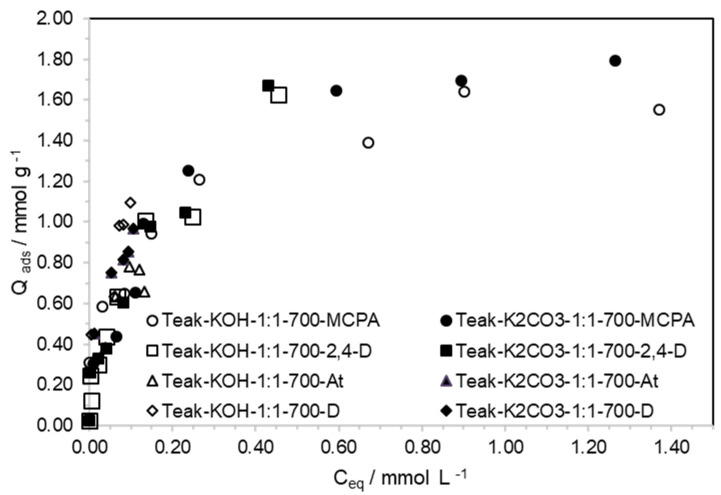
Adsorption of MCPA, 2,4-D, atrazine and diuron on ACs prepared from Teak, activated with KOH or K_2_CO_3_, at 973 K. (Teak-KOH-1-1-700 is the ACs designation and the last letters refer to the pesticides studied; At means atrazine and D means diuron.)

As shown in Figure 5, both ACs presented good performance in terms of the removal of the four pesticides from the aqueous phase. The MCPA and 2,4-D adsorption isotherm were, firstly, obtained from concentrated solutions, with concentrations varying from 0.125 to 1.25 mmol·L^−1,^ and from 0.113 to 1.13 mmol·L^−1^, respectively. In these conditions, the apparent maximum adsorption capacity for the MCPA was 1.64 and 1.65 mmol·g^−1^, on Teak-KOH-1-1-700 and Teak-K_2_CO_3_-1-1-700, respectively; and 1.63 and 1.67 mmol·g^−1^ for 2,4-D, on Teak-KOH-1-1-700 and Teak-K_2_CO_3_-1-1-700, respectively. The assays were very reproducible with a maximum standard deviation of 5 mg g^−1^ (≅0.03 mmol·g^−1^). When the adsorption assays were performed using solutions that contained 2.5 mmol·L^−1^ of MCPA, the maximum adsorption capacity attained was 1.88 mmol·g^−1^ on Teak-K_2_CO_3_-1-1-700.

From Figure 5, it is clear that the initial parts of the four isotherms overlap (Figure S5, Supplementary Materials), highlighting the existence of similar interactions between the four pesticides and AC surfaces. Over the entire concentration ranges evaluated for MCPA and 2,4-D, the differences between uptakes were not relevant.

Atrazine and diuron present very low water solubility at 298.15 K (30 and 42 mg·L^−1^, respectively). To create an objective comparison between the maximum amount of each pesticide removed from the liquid phase, dilute solutions of MCPA and 2,4-D were used to obtain data in similar conditions to those used with atrazine and diuron.

**Figure 6:**
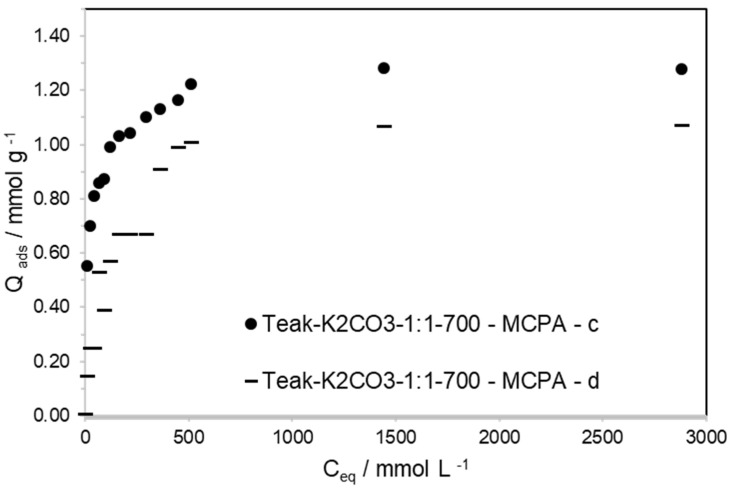
Kinetic study of adsorption of MCPA obtained from concentrated and dilute solutions.

In a second step, to prevent the same amount of pesticide being in contact with the ACs as that present in the concentrated solutions, the volume of solution used was increased to 175 mL, and the pesticide concentration was kept constant. In this case, 6 mg of AC was put in equilibrium in different volumes (from 25 to 175 mL) of each pesticide solution, with a concentration of 0.125 mmol·L^−1^. When the ACs were placed in different volumes of diluted solutions, the maximum adsorption capacity achieved was only 1.05 and 1.27 mmol·g^−1^ for MCPA on Teak-KOH-1-1-700 and Teak-K_2_CO_3_-1-1-700, respectively; and 0.65 and 0.62 mmol·g^−1^ for 2,4-D on Teak-KOH-1-1-700 and Teak-K_2_CO_3_-1-1-700, respectively. For the diluted solutions, the maximum amounts of MCPA and 2,4-D adsorbed by both ACs were far from the amount achieved using the concentrated solutions. When putting 175 mL of a solution with 0.125 mmol·L^−1^ in contact with 6 mg of AC, the number of moles of MCPA and 2,4-D in contact with each gram of AC was almost the same (3.65 mmol·g^−1^) as when putting 10 mg of AC in 25 mL of a solution with 1.25 mmol·L^−1^ (3.12 mmol·g^−1^). These results confirm that pesticide diffusion in the diluted solution is the key factor controlling the kinetics of the adsorption process and the maximum adsorption capacity, as presented in Figure 6.

In the initial parts of the isotherms, shown in Figure 5 and Figure 6 and Supplementary Materials, the amount of the four pesticides adsorbed was in the same range of values, independently of being obtained from concentrated or diluted solutions. Looking more closely, for the expansion of the initial part of the isotherm, seen in Figure 6, the amount of MCPA and 2,4-D adsorbed on Teak-K_2_CO_3_-1-1-700 was lower than that adsorbed on Teak-KOH-1-1-700. Teak-K_2_CO_3_-1-1-700 presents a mean pore size of 0.84 nm, and Teak-KOH-1-1-700 presents a mean pore size of 1.27 nm, which confirms the presence of pore size exclusion on Teak-K_2_CO_3_-1-1-700. Pore size exclusion was earlier referred to as taking place for pesticide removal only when the mean micropore diameter is smaller than 1.5 to 1.7 times the second widest dimension of the adsorptive [30]. However, the maximum amount of MCPA and 2,4-D adsorbed from solutions with a concentration higher than 1.13 mmol·L^−1^ on both ACs was very similar. This confirms that the pore-size exclusion phenomenon was not a relevant contribution when the adsorption of pesticides occurred using concentrated solutions, after a long equilibrium time (24 h).

The adsorption isotherms of the four pesticides obtained in Teak-KOH-1-1-700 and Teak-K2CO3-1-1-700 were analyzed based on the equations most commonly used for this purpose, i.e., Langmuir and Freundlich equations [39]. The values of the monolayer capacity (n_mL_), Langmuir constant (K_L_), and constant and exponent of Freundlich (K_F_ and n_F_) were obtained and are shown in Table 4.

Concerning the adsorption of MCPA and 2,4-D on Teak-KOH-1-1-700 and Teak-K_2_CO_3_-1-1-700, the n_L_ and K_F_ values were close but always slightly higher than the maximum adsorption capacity (n_max_) identified directly from the isotherms. This similarity confirms the good fitting of the Langmuir and Freundlich equations to these systems and also highlights that the maximum adsorption capacity of neither AC was achieved. Finally, in most pesticide-adsorbent systems, the adjustment was slightly better with the Langmuir equation.

Both ACs presented similar textural characteristics, i.e., surface area and microporous volume, and identical chemical characteristics, such as the percentage of N, H and S, and the pHpzc. Both ACs presented a basic character, with a pHpzc of 8.6 and 8.4 for Teak-KOH-1-1-700 and Teak-K_2_CO_3_-1-1-700, respectively. The maximum amount of each pesticide adsorbed on both ACs decreased by the following order: MCPA, 2,4-D, diuron and atrazine. This tendency can be attributed to the difference in solubility of pesticides in water and also their different pKa values.

Finally, the maximum amount of each pesticide adsorbed on both ACs was slightly higher than the values obtained previously on ACs prepared from Tectona Grandis sawdust, by physical activation with CO_2_, at 973 K (maximum adsorption capacity of MCPA on Teak-7480 was 1.56 mmol·g^−1^) [10]. Nevertheless, these data compare favorably with published results obtained on a diversity of commercial ACs used for this purpose [10,39,40].

## 4. Conclusions

ACs were prepared from Tectona Grandis waste by chemical activation with KOH and K_2_CO_3_, using different ratios between precursor/activating agent and different activating temperatures. The improved experimental conditions were a ratio of 1:1 between precursor/activating agent and a temperature of 973 K: these are recommended, for large-scale production of ACs. These conditions enable ACs, such as Teak-KOH-1-1-700 and Teak-K_2_CO_3_-1-1-700, to be obtained; these ACs performed very well in pesticide removal from the aqueous phase. Only a slight effect of the temperature was observed on the adsorption of MCPA, 2,4-D, atrazine and diuron, and the adsorption process was essentially exothermic.

The results presented are promising, allowing us to establish that Tectona Grandis sawdust is an excellent precursor for basic AC production. We can, therefore, expect good performances by these adsorbents in the removal of a broad range of pollutants from liquid streams. This achievement is very relevant for developing countries, such as East Timor, where Tectona Grandis sawdust is available and may constitute a source of income, creating an opportunity for the technical and industrial development of this region, and helping to leverage the circular economy.

## Figures and Tables

**Table 1:** Textural characteristics of ACs produced from Tectona Grandis sawdust, at different temperatures, by chemical activation with KOH. In Table 1 and Table 2, η is the AC production yield, A_BET_ is the apparent surface area, V_s_ is the volume total, A_s_ is the external surface area (V_s_ and A_s_ were obtained using the alfa-s method [31]), V_0_ is the volume of micropores, E_o_ is the adsorption energy and L_0_ is the mean pore size (V_0_, E_0_ and L_0_ were obtained using the Dubinin–Radushkevich equation [32].

Sample	ƞ/%	A_BET_/m^2^·g^−1^	α_s_		DR		
			A_s_/m^2^·g^−1^	V_s_/cm^3^·g^−1^	V_o_/cm^3^·g^−1^	E_o_/kJ·mol^−1^	L_o_/nm
Teak-KOH-1-2-700	17.5	709	65	0.30	0.17	18.6	1.35
Teak-KOH-1-1-700	16.7	995	57	0.43	0.26	19.9	1.27
Teak-KOH-2-1-700	24.1	337	36	0.20	0.09	14.9	3.05
Teak-KOH-1-1-600	20.4	581	21	0.20	0.15	25.4	0.77
Teak-KOH-1-1-450	25.0	170	27	0.10	0.04	14.9	3.10

**Table 2:** Textural characteristics of activated carbons produced from Tectona Grandis sawdust, at different temperatures, by chemical activation with K_2_CO_3_.

Sample	η/%	A_BET_/m^2^·g^−1^	α_s_		DR		
			A_s_/m^2^·g^−1^	V_s_/cm^3^·g^−1^	V_o_/cm^3^·g^−1^	E_o_/kJ·mol^−1^	L_o_/nm
Teak-K_2_CO_3_-1-2-700	20.2	1132	31	0.50	0.29	18.6	1.32
Teak-K_2_CO_3_-1-1-700	24.0	962	98	0.39	0.25	22.9	0.84
Teak-K_2_CO_3_-2-1-700	29.9	747	38	0.29	0.19	27.1	0.70
Teak-K_2_CO_3_-1-1-600	16.0	554	32	0.23	0.14	21.7	1.17
Teak-K_2_CO_3_-1-1-450	22.7	203	26	0.09	0.05	16.0	2.35

**Table 3:** Chemical characteristics of the ACs produced from Tectona Grandis sawdust, by chemical activation with KOH and K_2_CO_3_, at 973 K. (* O_2_ obtained by EDX).

Activated Carbon	N/wt%	C/wt%	H/wt%	S/wt%	O/* wt%	pH pzc
Teak-KOH-1-1-700	---	81.8	1.1	0.19	23.2	8.6
Teak-K_2_CO_3_-1-1-700	---	80.2	1.9	0.10	22.4	8.4

**Table 4:** Adsorption isotherm parameters of MCPA, 2,4-D, atrazine and diuron onto Teak-KOH-1-1-700 and Teak-K_2_CO_3_-1-1-700. n*_max_*, maximum adsorption capacity taken directly from the isotherm (mmol·g^−1^); n_mL_, monolayer capacity (mmol·g^−1^); K_L_, Langmuir constant (mmol·g^−1^); K_F_ (mmol·g^−1^ (dm^3^·mmol^−1^)^1/n^_F_ and n_F_ are Freundlich constant and exponent, respectively.

	System	Endo/exo	n_max_	n_mL_	K_L_	K_F_	n_F_
			mmol·g^−1^	mmol·g^−1^	mmol·g^−1^	mmol·g^−1^ (dm^3^·mmol^−1^)^1/n^_F_	
MCPA	Teak-KOH-1-1-700	Exo	1.64	1.84	6.6	1.72	2.9
	Teak-K_2_CO_3_-1-1-700	Exo	1.88	1.96	8.0	1.94	2.5
2,4-D	Teak-KOH-1:1-700	Exo	1.63	1.99	7.3	2.56	1.8
	Teak-K_2_CO_3_-1-1-700	Exo	1.67	1.66	8.4	2.44	1.8
Atrazine	Teak-KOH-1-1-700	Exo	0.78	0.99	22.4	1.55	3.2
	Teak-K_2_CO_3_-1-1-700	Exo	0.89	0.80	62.8	3.02	1.6
Diuron	Teak-KOH-1-1-700	Exo	1.10	1.19	49.6	2.71	2.6
	Teak-K_2_CO_3_-1-1-700	Exo	0.97	1.06	54.5	1.92	3.0

## Data Availability

More details can be found at Supplementary Materials.

## Supplementary Materials

The following supporting information can be downloaded at: https://www.mdpi.com/article/10.3390/ma15175842/s1, Figure S1: Nitrogen adsorption isotherms obtained on the ACs prepared from Teak, with KOH, at different temperature, Figure S2: Nitrogen adsorption isotherms obtained on the ACs prepared from Teak, with K_2_CO_3_, at different temperature, Figure S3: Microanalysis EDX spectra obtained on Teak-KOH-1-1-700, Figure S4: Microanalysis EDX spectra obtained on Teak-K_2_CO_3_-1-1-700, Figure S5: Adsorption of MCPA, 2,4-D, from diluted and concentrated solution, on ACs prepared from Teak, activated with KOH or K_2_CO_3_, at 973 K (Teak-KOH-1-1-700-MCPA-c; c means concentrated solutions, d- means diluted solutions).
