# Supplementary material for: Using Tectona Grandis Biomass to Produce Valuable Adsorbents for Pesticide Removal from Liquid Effluent
Source: Materials (Basel). 2022 Aug 24;15(17):5842. doi: 10.3390/ma15175842 (PMC9456658; doi:10.3390/ma15175842)
Supplement: Supplementary file 1 [file materials-15-05842-s001.zip › materials-1846862-supplementary.pdf]

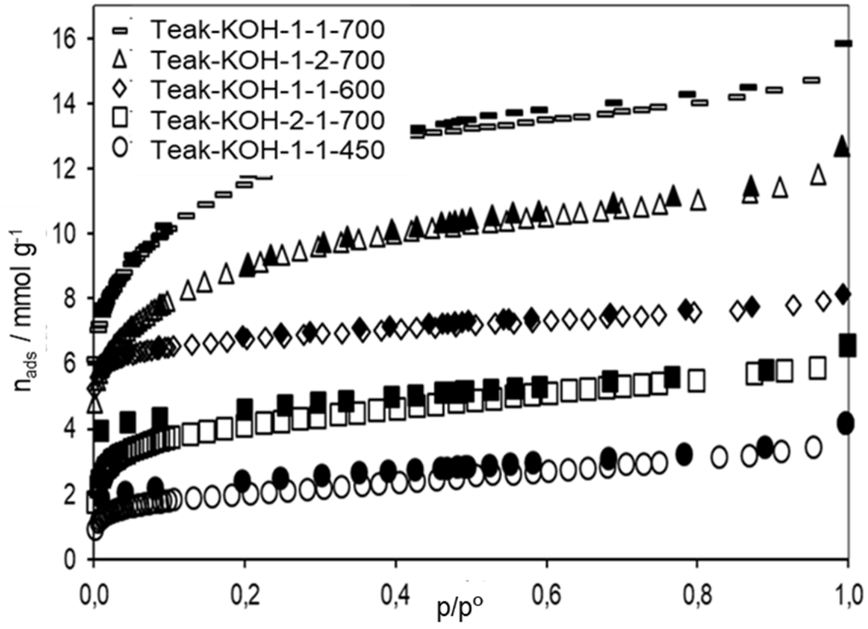

Figure S1. Nitrogen adsorption isotherms obtained on the ACs prepared from Teak, with KOH, at different temperature.

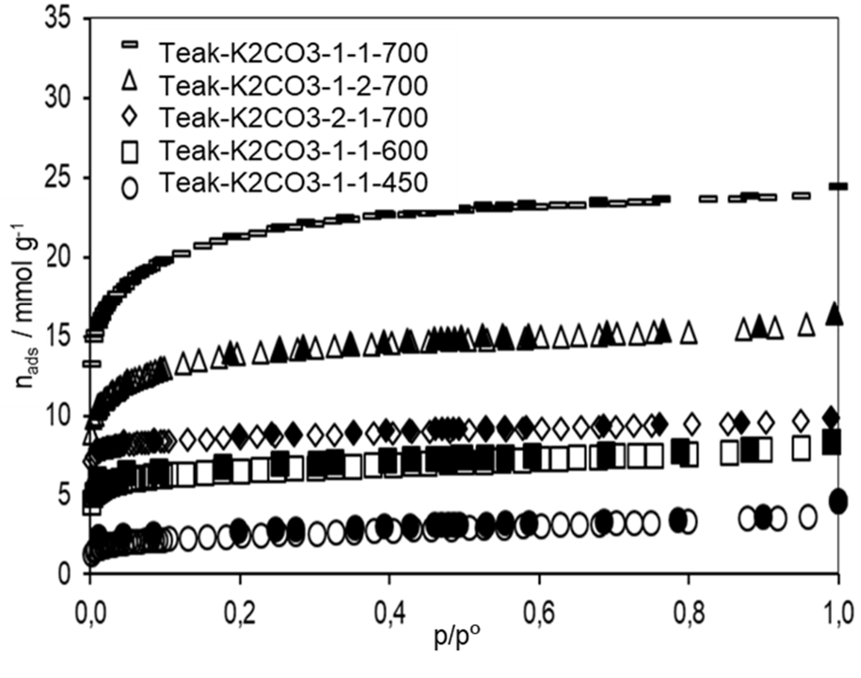

Figure S2. Nitrogen adsorption isotherms obtained on the ACs prepared from Teak, with K_2CO_3, at different temperature.

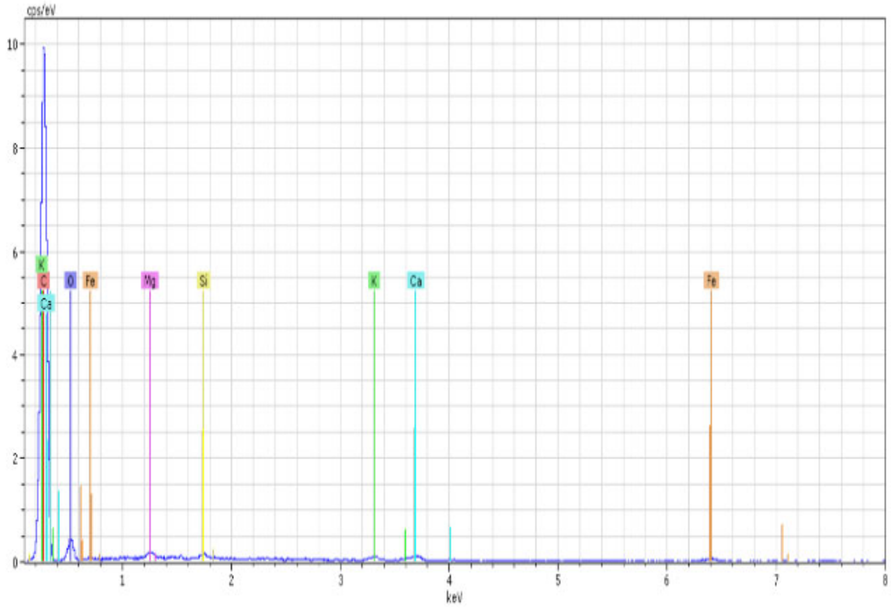

Figure S3. Microanalysis EDX spectra obtained on Teak-KOH-1-1-700.

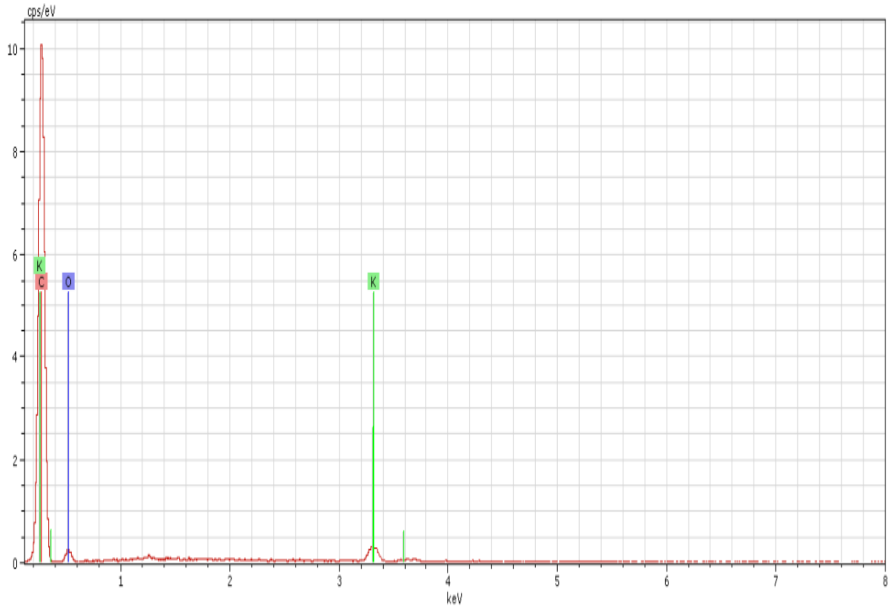

Figure S4. Microanalysis EDX spectra obtained on Teak-K_2CO_3-1-1-700.

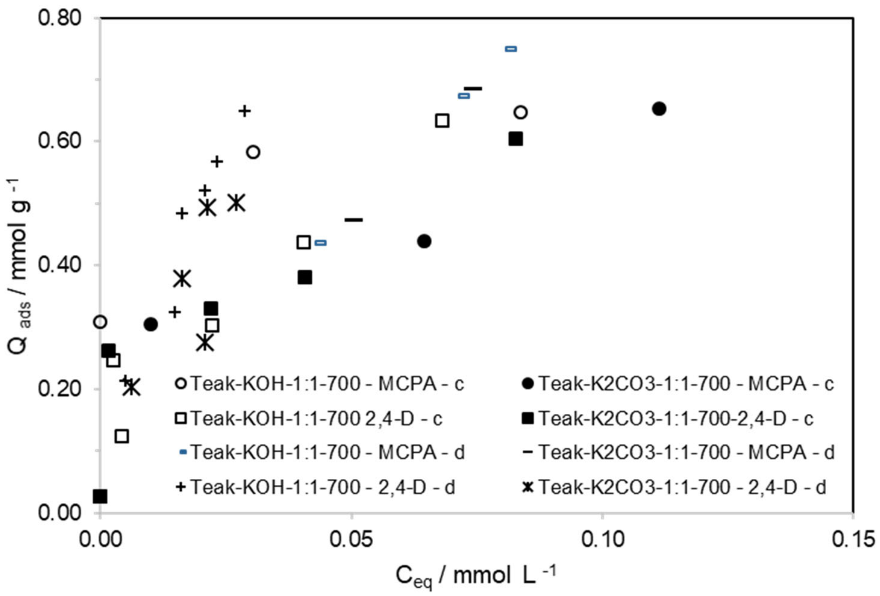

Figure S5. Adsorption of MCPA, 2,4-D, from diluted and concentrated solution, on ACs prepared from Teak, activated with KOH or K_2CO_3, at 973 K (Teak-KOH-1-1-700-MCPA-c; c means concentrated solutions, d- means diluted solutions).
